# Scalable and accurate multi-GPU-based image reconstruction of large-scale ptychography data

**DOI:** 10.1038/s41598-022-09430-3

**Published:** 2022-03-29

**Authors:** Xiaodong Yu, Viktor Nikitin, Daniel J. Ching, Selin Aslan, Doğa Gürsoy, Tekin Biçer

**Affiliations:** 1 Data Science and Learning Division, Argonne National Laboratory, 9700 Cass Avenue, Lemont, IL 60439 USA; 2 X-ray Science Division, Argonne National Laboratory, 9700 Cass Avenue, Lemont, IL 60439 USA; 3 Department of Electrical Engineering and Computer Science, Northwestern University, 2145 Sheridan Road, Evanston, IL 60208 USA

**Keywords:** Computational science, Computer science, Scientific data

## Abstract

While the advances in synchrotron light sources, together with the development of focusing optics and detectors, allow nanoscale ptychographic imaging of materials and biological specimens, the corresponding experiments can yield terabyte-scale volumes of data that can impose a heavy burden on the computing platform. Although graphics processing units (GPUs) provide high performance for such large-scale ptychography datasets, a single GPU is typically insufficient for analysis and reconstruction. Several works have considered leveraging multiple GPUs to accelerate the ptychographic reconstruction. However, most of these works utilize only the Message Passing Interface to handle the communications between GPUs. This approach poses inefficiency for a hardware configuration that has multiple GPUs in a single node, especially while reconstructing a single large projection, since it provides no optimizations to handle the heterogeneous GPU interconnections containing both low-speed (e.g., PCIe) and high-speed links (e.g., NVLink). In this paper, we provide an optimized intranode multi-GPU implementation that can efficiently solve large-scale ptychographic reconstruction problems. We focus on the maximum likelihood reconstruction problem using a conjugate gradient (CG) method for the solution and propose a novel hybrid parallelization model to address the performance bottlenecks in the CG solver. Accordingly, we have developed a tool, called PtyGer (**Pty**chographic **G**PU(multipl**e**)-based **r**econstruction), implementing our hybrid parallelization model design. A comprehensive evaluation verifies that PtyGer can fully preserve the original algorithm’s accuracy while achieving outstanding intranode GPU scalability.

## Introduction

Coherent diffraction imaging (CDI) is a lensless technique that can produce nanometer-scale resolution images by avoiding lens-imposed limitations of the traditional microscopy^1–7^. Ptychography^8^ is a scanning CDI technique that has gained popularity because of the increasing brilliance and coherence of the synchrotron light sources^9^. Ptychography benefits from the advantages of both CDI and scanning probe microscopy by using a focused beam of light (i.e., the probe) to scan the object at a series of overlapping scan positions and collecting the corresponding diffraction patterns at the far field by a pixelated detector. It can be extended to 3D by rotating the object to different view angles and repeating the scanning process at each view to yield a tomographic dataset^10^ or by using the new acquisition methods that are based on rotation as the “fast” scan axis^11^. Various image reconstruction models and iterative methods^12–19^ have been proposed to reconstruct the object based on the solutions of the phase retrieval problem. Since most of these approaches are optimized for single processing unit (GPU or CPU) and the measurement dataset sizes can reach terabytes scale, they can require a significant amount of processing time^20^. Meanwhile, the current ptychographic data acquisition instrument at Argonne’s Advanced Photon Source (APS) can yield 6 GB/second raw data from the detector. With the APS upgrade (APS-U) project, the anticipated data collection rate can even reach TB-per-second scale^21^, which enlarges the speed gap between the data collection instrument and the data processing component in the ptychography workflow. Therefore, it is crucial to improve the ptychographic image reconstruction performance in order to enable high-throughput ptychography experiments.

Over the past decade, GPUs have been broadly used for general-purpose computing because of their massive parallelism and computational power. Applications from various domains have been successfully accelerated with GPUs, including network intrusion detection^22–25^, biological sequence alignment^26,27^, program analysis^28,29^, and tomographic reconstruction^30–34^. Several works^35,36^ have implemented the standard algorithms for ptychographic reconstruction on a single GPU. However, the memory of a GPU still falls short compared with the data volumes of even a single view produced by ptychography experiments (see the section “Reconstruction efficiency”). Furthermore, the upcoming upgrade of the light source facilities will provide up to two orders of magnitude improvement in beam brightness, which will lead to a proportional increase in ptychography dataset sizes. These challenges motivate us to efficiently leverage multiple-GPU devices and therefore utilize aggregated memory to parallelize and accelerate ptychographic reconstruction.

Most of the current state-of-the-art high-performance computing systems are equipped as multinode multi-GPU machines^37–39^. Accelerating applications on these machines demands proper synchronizations and communications among the GPU devices. A few works^20,40,41^ implement ptychography on multi-GPUs with various reconstruction algorithms including ePIE^14^, relaxed averaged alternating reflections (RAAR)^42^, and difference map (DM)^15^. However, they all utilize only the Message Passing Interface (MPI) to handle the communications. MPI^43^, the de facto standard of internode communication handling, has been well established, and its functionality has been extended to be GPU-aware^44–46^. However, it has suboptimal performance for intra-node GPU-GPU communications because of factors such as the heavy overhead for the shared-memory model and the underutilization of the high-speed–low-speed mixed links^47–49^. Recently, NVIDIA has proposed the NVIDIA Collective Communications Library (NCCL)^50^ for multi-GPU communications. It provides lightweight and optimized multithreading solutions for handling intra-node GPU-GPU communications. Coupling NCCL with MPI can offer optimal performances for multi-GPU computing that requires both inter-node and intra-node communications.

In this paper we introduce a parallelization design for solving the maximum likelihood (ML) ptychographic reconstruction problem using a conjugate gradient (CG) solver^35^ on multi-GPU systems. Our design incorporates multithreaded GPU-GPU communication to enable the reconstruction of large views with aggregated GPU memories. CG solvers are known to converge faster than gradient-descent-based approaches (such as parallel ePIE) and are fully scalable. However, implementation of the CG solver on multi-GPU exposes challenges in preserving the full algorithmic equivalence because of the required scatter-gather operations (see the section “Challenges of CG solver parallelization”). To fit the multi-GPU CG solver, we propose a novel parallelization model in which we split each reconstruction iteration into four stages and then apply different communication patterns to different stages to accomplish various data transfer requests. This advanced design maintains the full equivalence of the original CG solver and minimizes the multi-GPU implementation overhead. We have implemented our solution in PtyGer, an openly available software package for **Pty**chographic **G**PU(multipl**e**)-based **r**econstruction. Our experiments demonstrate that PtyGer can provide reconstructions with remarkable multi-GPU scalability while maintaining the accuracy of the solution. In addition, PtyGer can be easily extended to 3D ptychography thanks to the data independence between the view angles and the tomo-ptycho joint solvers^10^. PtyGer also provides interfaces for other scalable algorithms that follow a similar data-processing pattern^15,16,51^ to be plugged into. Our contributions can be summarized as follows.

- We propose a fine-grained parallelization design for ptychographic reconstruction on multiple GPUs. We explore the challenges of parallelizing the CG-solver-based maximum likelihood phase retrieval algorithm and provide a novel hybrid model to tackle the challenges.
- We develop a ptychographic reconstruction software tool called PtyGer that implements our hybrid model design using Python and CUDA. It is scalable in terms of both dataset volume and GPU configuration.
- We extensively evaluate the efficacy and efficiency of PtyGer. We use both synthetic and real-world experimental datasets with various data volumes. The results show that PtyGer can provide accurate reconstruction with outstanding multi-GPU scalability.

## Related work

A variety of ptychographic reconstruction algorithms have been proposed during the past two decades. The ptychographic iterative engine (PIE)^12,13^ is an iterative phase retrieval approach employing the diffraction patterns as a known illumination function. It demonstrates fast convergence rate^52,53^. However, it lacks robustness when noise is present in the measurements. Moreover, the PIE and its variants (ePIE^14,54^, 3PIE^55^) are inherently sequential since they have to update probe and object images after processing each diffraction pattern. In contrast to the PIE family, some reconstruction algorithms are parallelizable^56^, including the DM algorithm^15,16^ and the RAAR algorithm^42^. Another notable parallel-friendly ptychographic reconstruction approach uses the ML model^18,19^ and is derived from the cost-function optimization technique^17^. The DM and RAAR algorithms and ML-based models that use the CG solver are all naturally parallelizable since they need to update only the estimated object image after simultaneously processing a set of diffraction patterns. Recently, some advanced techniques^10,57^ have been proposed to further refine the reconstructed images.

Several works discuss the GPU-based implementations of ptychography. PyNX^58^ implements the DM algorithm on the GPU using the OpenCL language. PtychoShelves^36^ provides a MATLAB-based GPU reconstruction engine that supports the DM and ML algorithms. However, both of them allow reconstructions only on a single GPU. Marchesini et al. developed a multi-GPU-based ptychographic solver called SHARP^41^ implementing the RAAR algorithm. Dong et al. implemented the DM algorithm on multiple GPUs^20^. But the methods utilize only MPI to handle both intra- and internode multi-GPU processing and thus are suboptimal. Although CUDA-aware MPI enables direct GPU-GPU data access and transfer, MPI is not optimized for the intranode multi-GPU communications because of inefficient utilization of high-speed links (e.g., NVLink). PtychoLib^40^ proposes a fine-grained design for multi-GPU-based ptychography. It parallelizes the ePIE by simultaneously processing all diffraction patterns and updating the reconstructed image only once in each iteration. However, this parallelization does not comply with the design principle of ePIE and can introduce artifacts while reconstructing experimental data. It also suffers from the suboptimal performance of MPI for intranode GPU-GPU communications. Recently, PtychoLib is integrated into PtychoPy^21^, which is a GPU framework for the entire ptychographic data analysis workflow. PtychoPy implements ePIE, DM, and ML-based models with Least-Square (LS) solver. However, currently, only its ePIE implementation supports the execution on multi-GPU environment.

The difficulty of scaling applications on multi-GPU configurations is often underestimated. Simply distributing the workloads and duplicating the single-GPU procedure onto other GPUs usually lead to incorrect results and poor scaling. Therefore, implementations on multi-GPU configurations require design considerations for communications via mixed topology containing both PCIe and NVlink, asynchronous execution-communication, and so on. A limited number of studies focus on designing fine-grained multi-GPU-based implementations. BLASX^59^ is a library providing high-performance Level 3 BLAS primitives for multi-GPU-based linear algebra. Gunrock^60^ and Groute^61^ are two frameworks that support multi-GPU graph analytics. MAPS-Multi^62^ and Blink^63^ propose designs for multi-GPU-based machine learning. All these works optimize multi-GPU performance by carefully balancing the application workload and tuning GPU peer-to-peer (P2P) communication while considering both algorithmic and multi-GPU architectural characteristics. They verify that the fine-grained parallelization is essential to fully unleash the multi-GPU’s computing power for accelerating real-world applications.

## Results

We evaluate both the efficacy and efficiency of our CG-solver-based PtyGer on a multi-GPU platform. This platform includes two Intel Xeon Silver 4116 CPUs (host) and eight NVIDIA GeForce RTX 2080Ti GPUs (devices). The host has 768 GB of total memory, and each CPU consists of 12 physical cores (or 24 cores in total for a host). Each 2080Ti card is built on the latest Turing microarchitecture and integrates 68 streaming multiprocessors (SMs) with 64 CUDA cores per SM, hence totally holding 4352 CUDA cores on chip. Each card has a 11 GB off-chip global memory that is shared by all SMs. All 68 SMs also share a 5.5 MB on-chip L2 cache, and each SM has 64 KB of private L1 cache (shared memory). The GPUs are interconnected by using PCIe that are capable of 11 GB/s unidirectional bandwidth. The Turing microarchitecture disables P2P communication via PCIe; hence the GPU-GPU data transfers are accomplished via host memory. Leveraging direct GPU-GPU communication with application-specific design potentially can further improve the overall performance^64^.

We evaluate our tool using three datasets: *siemens-star*^65^, *coins*, and *pillar*. The first two datasets are synthetic, produced by generating the diffraction patterns from a test image (*siemens-star*) and a real image (*coins* (https://commons.wikimedia.org/wiki/File:Kiloware.JPG)), respectively. The third dataset is a real experimental dataset from an integrated circuit sample; the data was collected at the 2-ID-D microscopy beamline at the Advanced Photon Source with 0.2 ${\upmu \!\hbox {m}}$ $\times$ 0.2 ${\upmu \!\hbox {m}}$ beam size (https://www.aps.anl.gov/Microscopy/Beamlines/2-ID-D). The dimensions of the reconstructed images are $2048\times 2048$, $1024\times 1024$, and $1024\times 768$, respectively. The probe dimensions are $256\times 256$ for all datasets. We introduce different computation demands for reconstructions by subsampling each of the datasets to 4K, 8K, and 16K diffraction patterns. The total number of iterations is set to 128 for all reconstructions.

### Reconstruction efficacy

In this section we evaluate the efficacy of PtyGer by examining the accuracy of the reconstructions. For each dataset we calculate the structural similarity (SSIM)^66^ and peak signal-to-noise ratio (PSNR) between the reconstructed image and the reference image (ground truth) after each iteration: 1 in SSIM indicates perfect structural similarity while a value larger than 50 dB in PSNR typically represents that the difference between two images is tiny. Since the real experimental data have no reference object image, we evaluate the reconstruction results only of synthetic datasets.

**Figure 1:**
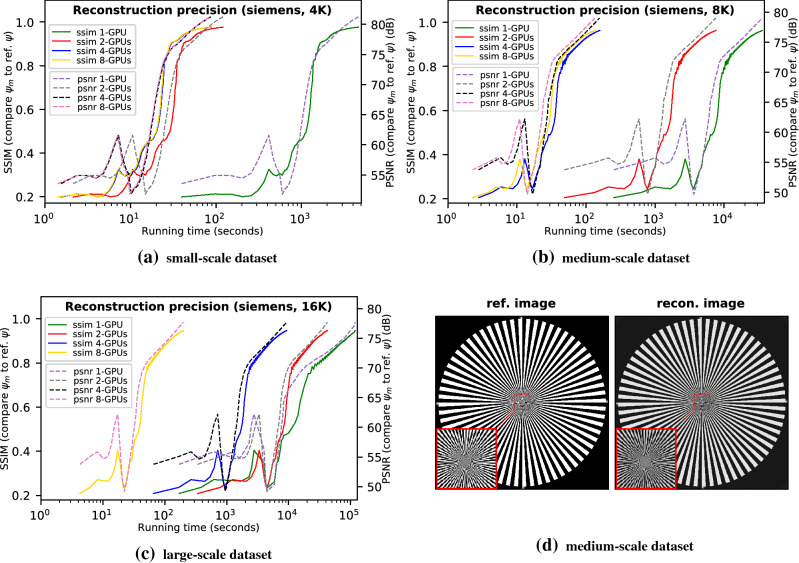
Reconstruction accuracy evaluations using (**a**) small-scale (4K diffraction patterns), (**b**) medium-scale (8K), and (**c**) large-scale (16K) synthetic *siemens-star* datasets. For each evaluation, we calculate both the SSIM and PSNR between the reconstructed image and the reference image after each iteration. We use the running time as the x-axis to distinguish the results of different GPU configurations. We note that in each subfigure the reconstructions with different number of GPUs actually have the same convergence curve. The visual difference is caused by different speeds of running a iteration. (**d**) shows the comparison between the 4-GPU reconstructed image of the 8K dataset and the reference image.

Figure 1 demonstrates the reconstruction accuracy of PtyGer on *siemens-star* datasets using 8-GPU, 4-GPU, and 2-GPU configurations, respectively. It also includes the single-GPU reconstruction result^35^ as the counterpart. In each subfigure the x-axis indicates the running time of PtyGer in log-10 scale, while the left-hand y-axis indicates the SSIM and the right-hand y-axis represents PSNR, both between the reconstructed image and the reference image. Figure 1a shows the results on the small-scale *siemens-star* dataset (4K diffraction patterns). It indicates that although taking different amounts of time, the SSIMs of all multi-GPU configurations approach the reference image while the PSNRs start at around 55 dB and reach above 80 dB at the end. We also observe that the multi-GPU reconstructions have similar convergence curves compared with the single-GPU based reconstruction. More specifically, after approximately 65 iterations, the multi-GPU-based reconstructions have their SSIM and PSNR going beyond 0.95 and 75 dB and monotonically increasing toward the reference image thereafter. Figure 1b and c show the results of the medium-scale *siemens-star* dataset (8K) and large-scale *siemens-star* dataset (16K), respectively. Similarly, they indicate that multi-GPU-based reconstructions on both datasets have their SSIM converging to 1 and PSNR reaching 80 dB and expose the same convergence curves compared with their single-GPU based counterparts. Figure 1d compares the actual reconstructed image of the 8K dataset using 4 GPUs with the original siemens-star image. The zoom-in to the center of the images highlights that the hybrid parallelization model and the subimage alignment in PtyGer introduce no artifacts. Although reconstructions with different GPU configurations have the same precision, they cost different amounts of time to converge. The convergence speed is determined mainly by the per GPU workload that can fit into the GPU memory. For example, while 4K diffraction patterns fit in 2-, 4-, and 8-GPU configurations, the 1-GPU configuration cannot accommodate all the data structures and therefore shows significant slowdown compared with the other configurations. Similarly, for 16K diffraction patterns, the dataset can fit only into the 8-GPU configuration, which shows an execution time consistent with 4K diffraction patterns’ dataset. However, all the other configurations show performance degradation due to memory limitations. We will comprehensively evaluate the converging efficiency of PtyGer in the next section.

**Figure 2:**
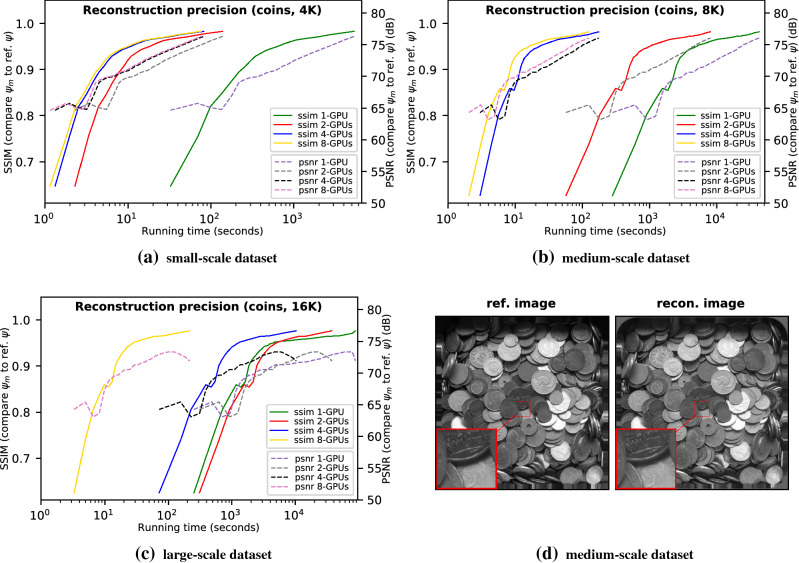
Reconstruction accuracy evaluations using (**a**) small-scale (4K diffraction patterns), (**b**) medium-scale (8K), and (**c**) large-scale (16K) synthetic *coins* datasets. For each evaluation, we calculate both the SSIM and PSNR between the reconstructed image and the reference image after each iteration. We use the running time as the x-axis in order to distinguish the results of different GPU configurations. We note that in each subfigure the reconstructions with different numbers of GPUs actually have the same convergence curve. The visual difference is caused by different speeds of running an iteration. (**d**) shows the comparison between the 4-GPU reconstructed image of the 8K dataset and the reference image.

Figure 2 demonstrates another set of reconstruction accuracy evaluations using the same experimental design as Fig. 1 on small-scale *coins* (Fig. 2a), medium-scale *coins* (Fig. 2b), and large-scale *coins* (Fig. 2c) datasets, respectively. The SSIM and PSNR in Fig. 2 indicate that the reconstructions of *coins* datasets with different multi-GPU configurations all converge to the reference image and the convergence curves are identical to their corresponding single-GPU-based counterparts’ patterns. Figure 1d displays the actual 4-GPU reconstructed image of the 8K dataset and compares it with the original coins image. We note that no artifacts are observed at the zoomed-in centers of the images, where the GPUs exchange border information.

These evaluation results show that our PtyGer tool with various numbers of GPUs can fully preserve the functional equivalence compared with the original algorithm implemented on a single GPU. The outputs of our multi-GPU-based reconstructions are mathematically similar enough to the ground truths (the reference images), and the convergence trends are identical to the original single-GPU-based implementation.

### Reconstruction efficiency

In this section we evaluate Ptyger’s reconstruction efficiency, that is, its speedup compared with that of the single-GPU counterpart^35^ (the baseline). The preceding subsection verified that PtyGer preserves the algorithmic equivalence; hence, comparing the convergence speeds of PtyGer with the counterpart can indicate the performance speedups. We measure the convergence speed by recording the least-square norm (2-norm) of the difference between current and previous iterations’ outputs (i.e., $\psi _{m}-\psi _{m-1}$). 2-norm being 0 represents perfect convergence. In practice, when the 2-norm goes below a certain threshold, the reconstruction result is considered converged. Notice that our experiments use a fixed number of iterations (128) instead of a stop criterion; hence the reconstructions keep iterating even if the outputs are converged. Therefore, the 2-norms can decrease further or fluctuate within a small range at the end. We evaluate PtyGer’s efficiency on all nine datasets.

**Figure 3:**
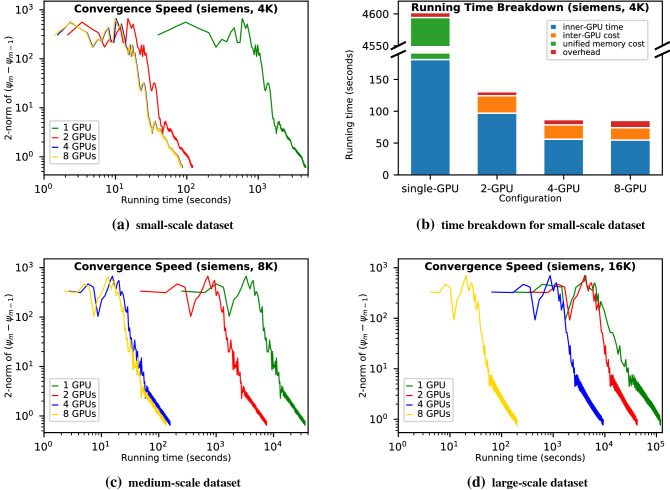
Convergence speed evaluations using (**a**) small-scale (4K diffraction patterns), (**c**) medium-scale (8K), and (**d**) large-scale (16K) synthetic *siemens-star* datasets. For each evaluation, we apply various GPU configurations (single-GPU, 2-GPU, 4-GPU, and 8-GPU) and compute the difference of the reconstructed images before (i.e., $\psi _{m-1}$) and after (i.e., $\psi _{m}$) the update stage in each iteration by measuring the pixels’ 2-norm. Notice that we use a log-10 scale for both axes. (**b**) provides more details for (**a**) by breaking down the running time. In the single-GPU case, notice that there is no inter-GPU cost; we use a broken bar for unified memory cost for better illustration.

Figure 3 demonstrates the reconstruction convergence rates of three *siemens-star* datasets using 8-, 4-, 2-, and 1-GPU configurations. In each subfigure, the x-axis indicates the running time and the y-axis indicates the 2-norm. We emphasize that both axes are in log-10 scale. Consequently, the lower bound of the 2-norm is negative infinity (i.e., 0 in regular scale). Figure 3a shows the efficiency comparisons of reconstructions on the small-scale *siemens-star* dataset (4K diffraction patterns). Figure 3b breaks down the running time under each configuration. We use a broken bar to demonstrate the outlier (i.e., single-GPU). As indicated in both figures, the single-GPU-based reconstruction takes approximately 1.3 hours. This slow execution is caused by the fact that the size of the dataset exceeds the memory capacity of a single GPU. We note that the CG-solver yields a large memory footprint during the execution due to buffering the intermediate results. Instead of aborting the execution, the reconstruction leverages CUDA’s unified memory to handle the excess data. It provides the unified virtual address space to the GPU and CPU memories, so the program can automatically utilize the CPU memory to assist the GPU execution. However, this technique has a large overhead. As shown in Fig. 3b, unified memory cost (green segment) dominates the single-GPU execution time (95.8%). The cost includes expensive CPU-GPU data migrations and page fault handlings occurring at each CUDA arithmetic operation. Figure 3a shows that the 2-GPU-based reconstruction is 38.6$\times$ faster than the original single-GPU reconstruction. The speedup is *superlinear* because the splitting of the dataset makes the workload of each GPU (2K diffraction patterns per GPU) fit into GPU memory, and hence the 2-GPU reconstruction avoids host-device communication. Figure 3b shows that inner GPU computing (blue segment) takes the majority (74.2%) of the 2-GPU reconstruction time. One other main component (20.8%) is the inter-GPU cost (orange segment) including GPU-GPU synchronizations and communications. The one-time overhead (red segment) consisting of data preparation and device management is minor (5%). Figure 3a also shows that the 4-GPU-based reconstruction has 1.5$\times$ speedup compared with that of the 2-GPU reconstruction. We observe that in Fig. 3b, although the inner-GPU computing of the 4-GPU is nearly 2$\times$ faster because of the halved per GPU workload, the overall speedup is sublinear. The main reason is that since more devices are involved in the computation, the inter-GPU cost and one-time overhead are slightly larger than for the 2-GPU.

Figure 3a shows the 8-GPU reconstruction has nearly the same efficiency as the 4-GPU configuration. This is because increasing the number of GPUs makes each GPU’s workload (0.5K diffraction patterns) too small to efficiently occupy the GPU computing resources. Figure 3b shows that both 4-GPU and 8-GPU configurations are memory bound since they have similar computation times.

Figure 3c and d show the reconstruction efficiency comparisons on the medium-scale *siemens-star* dataset and large-scale *siemens-star* dataset, respectively. In Fig. 3c, we observe that even though running faster than the single-GPU configuration, the 2-GPU reconstruction nevertheless takes a long time since the divided workload on each GPU (4K per GPU) still exceeds the GPU memory capacity. We also observe that the 4-GPU configuration is 42.4$\times$ faster than the 2-GPU. The speedup is superlinear for the same reason mentioned before. The 8-GPU-based reconstruction is 1.3$\times$ faster than the 4-GPU. Compared with the 8-GPU reconstruction on the small-scale *siemens-star* dataset, the reconstruction on the medium-scale dataset doubles the workload per GPU and therefore better utilizes the computing resources. Thus, for the medium-scale dataset, increasing the number of GPUs from 4 to 8 can improve the overall performance. Figure 3d shows that for the large-scale *siemens-star* dataset, even the 4-GPU configuration has insufficient memory. On the other hand, the 8-GPU configuration makes the per GPU workload small enough to fit into the GPU memory and achieves superlinear speedup (41.5$\times$) compared with the 4-GPU configuration.

**Figure 4:**
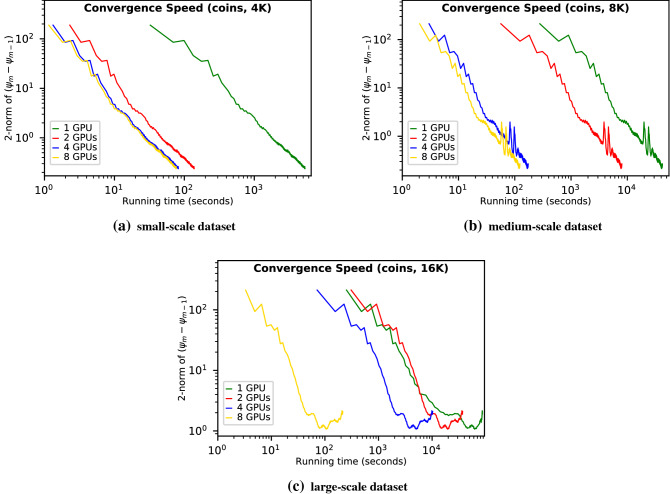
Convergence speed evaluations using (**a**) small-scale (4K diffraction patterns), (**b**) medium-scale (8K), and (**c**) large-scale (16K) synthetic *coins* datasets. For each evaluation, we apply various GPU configurations (single-GPU, 2-GPU, 4-GPU, and 8-GPU) and compute the difference of the reconstructed images before (i.e., $\psi _{m-1}$) and after (i.e., $\psi _{m}$) the update stage in each iteration by measuring the pixels’ 2-norm. Notice that we use a log-10 scale for both axes.

**Figure 5:**
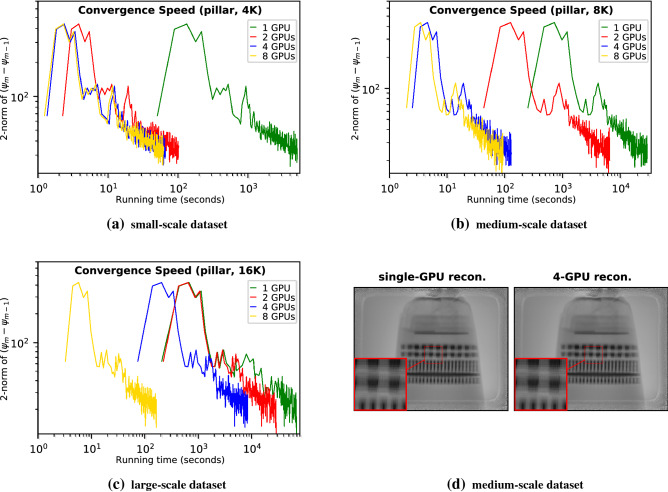
Convergence speed evaluations using (**a**) small-scale (4K diffraction patterns), (**b**) medium-scale (8K), and (**c**) large-scale (16K) real-beamline *pillar* datasets. For each evaluation, we apply various GPU configurations (single-GPU, 2-GPU, 4-GPU, and 8-GPU) and compute the difference of the reconstructed images before (i.e., $\psi _{m-1}$) and after (i.e., $\psi _{m}$) the update stage in each iteration by measuring the pixels’ 2-norm. Notice that we use a log-10 scale for both axes. (**d**) compares the 4-GPU reconstructed image of 8K dataset with the single-GPU result.

Figures 4 and 5 show another two sets of efficiency comparisons on *coins* and *pillar* datasets, respectively. As shown in Figs. 4a and 5a, for the small-scale datasets, the workloads exceed the capacity of a single-GPU memory ;hence the single-GPU reconstruction is very slow. The 2-GPU configurations accommodate the per GPU workload within the GPU memory capacity and thus achieve superlinear speedups (38.4$\times$ in Fig. 4a and 39.7$\times$ Fig. 5a compared with the single-GPU one). Increasing the number of GPUs from 2 to 4 can achieve additional 1.7$\times$ and 1.6$\times$ speedups. However, further increasing the number of GPUs from 4 to 8 shows no improvement because of the underutilized GPU resources. For the medium-scale datasets, the 2-GPU configuration cannot keep the per GPU workload within the GPU memory capacity; hence the 4-GPU configuration shows 40.2$\times$ (Fig. 4b) and 42.4$\times$ (Fig. 5b) superlinear speedups. The 8-GPU configuration can further improve the efficiency by 1.4-fold (Fig. 4b) and 1.4-fold (Fig. 5b) compared with the 4-GPU. For the large-scale datasets, the per GPU workload exceeds the GPU memory capacity even with four GPUs, but the 8-GPU reconstructions can still afford the large workloads and achieve superlinear speedups (42.9$\times$ (Fig. 4c) and 43.1$\times$ (Fig. 5c), respectively) over the 4-GPU configurations. Figure 5d displays the reconstructed pillar image with 8K diffraction patterns using the 4-GPU configuration. Since there is no reference pillar image, we compare the 4-GPU reconstructed image with the single-GPU result. The comparison shows that scaling the reconstruction of real beamline data to multiple GPUs using PtyGer preserves the reconstructed image’s quality. Focusing on the image centers shows that there are no artifacts at the overlapped regions among the GPUs. All these comparisons verify that our multi-GPU PtyGer can successfully increase the overall reconstruction efficiency. The rate of increase is affected by both the dataset size and the number of GPUs. If the workload exceeds the GPU memory capacity, then adding more GPUs can achieve superlinear speedup since the aggregated GPUs memory decreases the host-device memory operations. Once the workload fits into aggregated memory, adding more GPUs can still improve the reconstruction efficiency; however, the performance improvement is sublinear because of inter-GPU synchronization and communication overheads. However, once the per GPU workload is significantly insufficient to occupy the GPU computing resources, the performance gain stops growing.

### Comparisons with existing software

Conducting fair performance comparisons between PtyGer and the other ptychographic reconstruction software are difficult, due to a) the different reconstruction algorithms that are adopted in different software, b) some software are not publicly available. In this paper, we compare PtyGer to two open-source counterparts: PtychoShelves^36^ and PtychoPy^21^. The former currently does not support multi-GPU execution. In the case of exceeding the single-GPU memory capacity, it groups the dataset and processes the subsets in sequence. The latter supports multi-GPU execution only when using ePIE algorithm. Although both counterparts provide multiple options of the reconstruction algorithms, neither of them implement the CG solver for the ML-based models. To make the comparison as fair as possible, we choose Least-Square (LS) solver in PtychoShelves for ML-based models and ePIE algorithm in PtychoPy for multi-GPU execution. We note that, in our comparisons, we emphasize the scalability rather than the absolute values of reconstruction time. It is because the time is majorly determined by the efficiency of employed reconstruction algorithm which is off-topic of this paper.

We run all the three software on the same multi-GPU platform to reconstruct the dataset with 4K diffraction patterns. PtychoShelves groups the dataset into multiple subsets and process them one by one in a single GPU. For PtychoPy and our PtyGer, we perform the reconstructions with 1-, 2-, 4-, and 8-GPU configurations. On a single GPU, PtyGer shows 54.8$\times$ and 29.2$\times$ slower reconstructions compared to PtychoShelves and PtychoPy, respectively, since the CG solver yields significant larger memory footprint than other solvers and algorithms. When the GPU number increases to two, although PtyGer is still slower than the counterparts, we observe that the performance gap dramatically shrinks to 1.38$\times$ and 1.03$\times$ since PtyGer gets rid of the huge unified memory overhead with two GPUs. When we further increase the number of GPUs from 2 to 4, PtyGer starts to exhibit better performance than the counterparts with ratios at 1.19$\times$ and 1.31$\times$. This is because PtychoShelves has no scalability and PtychoPy shows only around 1.3$\times$ scalability, whereas our PtyGer shows around 1.6$\times$ scalability along with the GPU device scale-up. PtychoPy demonstrates less scalability than PtyGer since it uses MPI to handle intra-node GPU-GPU communications, which incurs additional overhead. There is no further performance improvement with 8-GPU due to resource underutilization, but PtyGer remains outperforming the counterparts. Based on the above comparisons, we can conclude that PtyGer has better scalability than the existing ptychographic reconstruction software. We note that our PtyGer’s parallelization methodology is also applicable to other solvers with necessary solver-specific adaptations.

### Summary and discussion

We comprehensively evaluate our PtyGer using both synthetic and real beamline data with various sizes. The evaluation shows that PtyGer fully preserves the accuracy of the original sequential algorithm. We also show great scalability efficiency with PtyGer, in some cases superlinear, with the increasing numbers of GPUs and large workloads. The multi-GPU reconstructions are significantly faster than the single-GPU counterparts because of the elimination of unified memory usage (host-device data movement) and aggregated GPU memory. When the per GPU workload fits into the GPU memory, increasing the device number by a factor of two results in up to 1.7$\times$ performance improvement. The sublinearity is caused by the unavoidable preprocessing overhead and inter-GPU costs. Our hybrid parallelization model design has significantly reduced the inter-GPU communication.

The optimal number of GPU devices depends on the dataset size. More GPUs do not necessarily provide better performance. When the per GPU workload is too small, the system reaches the memory I/O bound. Therefore, adding more GPUs will not increase the reconstruction speed and can lead to a waste of the computational resources. Empirically, using 2 GPUs for a small dataset can achieve the best resource utilization; 4 GPUs are adequate for a medium dataset; 8 GPUs are needed for a large dataset. Whenever a sufficient number of GPUs is utilized for a given workload, any excess GPUs can be used for reconstructing other view angles in 3D ptychography.

## Methods

### Background

In this section we provide the background information about the ptychography model, the iterative phase retrieval algorithm, and the gradient-based solvers.

#### Forward problem of ptychography

**Figure 6:**
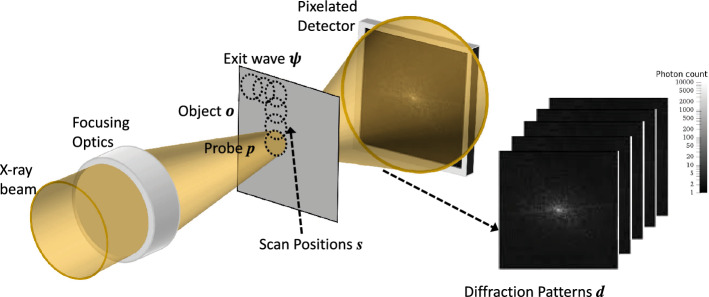
Typical setup of ptychography data collection. The focused probe *p* raster-scans the object *o* at overlapped positions *s*. A series of diffraction patterns of the exit waves are collected by the pixelated detector to form the diffraction data *d*.

Figure 6 schematically shows a generic ptychography setup. In ptychography, a focused coherent x-ray beam of light (i.e., probe *p*) is used to scan the object *o*, and the diffraction patterns of the transmitted radiation are collected via a pixelated photon counting detector. The probe scans the object in a series of consecutive and overlapped illumination positions (i.e., scan position *s* in Fig. 6). Accordingly, the detector collects a series of diffraction patterns denoted by *d*. Ptychography can be mathematically described by the forward model from exit wave $\psi$ to diffraction pattern *d*:

1 $$\begin{aligned} |{{\,\mathrm{\mathscr {G}}\,}}\psi |^2 = |{{\,\mathrm{\mathscr {F}}\,}}{{\,\mathrm{\mathscr {Q}}\,}}\psi |^2 = d, \end{aligned}$$

where ${{\,\mathrm{\mathscr {G}}\,}}$ is the ptychography operator that represents applying Fourier transforms ${{\,\mathrm{\mathscr {F}}\,}}$ to the diagonal operator ${{\,\mathrm{\mathscr {Q}}\,}}$. ${{\,\mathrm{\mathscr {Q}}\,}}$ acts as an elementwise multiplication of probe *p* and exit wave $\psi$ at all scan positions.

#### Inverse problem for ptychography

In this section we briefly recapitulate our approach for solving the 2D ptychography problem. For details we refer to our former papers^10,35^, where we considered maximum likelihood and least-squares (LS) estimates for the solution. As an example, here we consider the ML estimate formulation, although all the techniques introduced in this paper are also applicable to the LS estimator.

The exit wave $\psi$ can be numerically reconstructed based on the solution of the phase retrieval problem. Photon collection of the detector is a Poisson process^67^, and the probability of acquiring data $d_j$, $j=1,2,\dots ,n$, is given by the likelihood function.

The corresponding ML estimate of $\psi$ then can be computed by solving the following minimization problem:

2 $$\begin{aligned} \begin{aligned} F(\psi )=\sum _{j=1}^{n} \left\{ |{{\,\mathrm{\mathscr {G}}\,}}\psi |_j^2-2d_j\log |{{\,\mathrm{\mathscr {G}}\,}}\psi |_j \right\} \rightarrow \min , \end{aligned} \end{aligned}$$

for which the gradient is given as follows:

3 $$\begin{aligned} \nabla _\psi F(\psi ) ={{\,\mathrm{\mathscr {G}}\,}}^H\left( {{\,\mathrm{\mathscr {G}}\,}}\psi -\frac{d}{({{\,\mathrm{\mathscr {G}}\,}}\psi )^*}\right) , \end{aligned}$$

where ${{\,\mathrm{\mathscr {G}}\,}}^H={{\,\mathrm{\mathscr {Q}}\,}}^H{{\,\mathrm{\mathscr {F}}\,}}^H$, ${{\,\mathrm{\mathscr {Q}}\,}}^H$ operates as an elementwise multiplication by the conjugate of probe *p*, and ${{\,\mathrm{\mathscr {F}}\,}}^H$ is the inverse Fourier transform.

With the gradient we can iteratively reconstruct the object image using various solvers. We can use the gradient descent (GD) solver to reconstruct the image^10^. In this case iterations of updating the estimated object can be written as follows:

4 $$\begin{aligned} \psi _{m+1} = \psi _{m} - \gamma \nabla _\psi F(\psi ), \end{aligned}$$

where $\gamma$ is given as a constant small step length. The GD solver has a slower convergence rate compared with the conjugate gradients family of solvers^68–71^. In our former work the CG solver with the Dai–Yuan conjugate direction^68^ demonstrated a faster convergence rate in 2D ptychography compared with analogues. The iterations for object updating with the Dai–Yuan direction can be written as

5 $$\begin{aligned} \psi _{m+1} = \psi _m + \gamma _m \eta _m, \end{aligned}$$

where $\gamma _m$ is the step length and $\eta _m$ is the search direction given by the recursive Dai–Yuan^68^ formula:

6 $$\begin{aligned} \begin{aligned} \eta _{m}= -\nabla _\psi F(\psi _{m})+\frac{\left\Vert \nabla _\psi F(\psi _{m})\right\Vert _2^2}{ \langle \eta _{m-1},{\nabla _\psi F(\psi _{m})}-{\nabla _\psi F(\psi _{m-1})\rangle }}\eta _{m-1} \end{aligned} \end{aligned}$$

with the initial direction as the steepest descent direction, $\eta _0=-\nabla _\psi F(\psi _0)$. $\langle a,b\rangle$ is defined as $\sum _i^z a^*_i b_i$, and *z* is the number of pixels of object $\psi$. The step length $\gamma _m$ is computed through a line search procedure^72^. Line search starts at an initial large step length and repeatedly shrinks it until the following is satisfied:

7 $$\begin{aligned} F(\psi _m+\gamma _m\eta _m) \leqslant F(\psi _m) + \gamma _m t, \end{aligned}$$

where *t* is a constant termination parameter usually set to 0 in practice.

**Figure 7:**
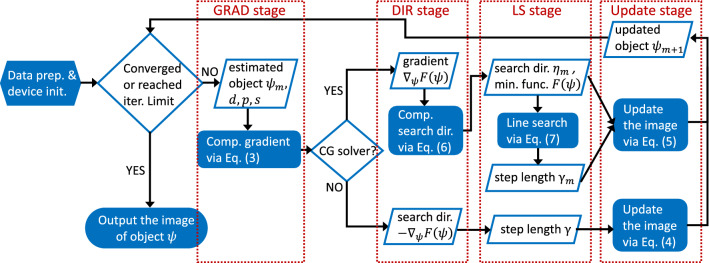
Flowchart of iterative maximum likelihood phase retrieval algorithm using the gradient-based solver. Each iteration can be divided into four stages: *GRAD stage*, *DIR stage*, *LS stage*, and *update stage*.

The complete iterative solution of the ptychographic reconstruction can be summarized with the workflow shown in Fig. 7. In the workflow, an iteration is divided into four stages, where we apply different parallelization schemes to different stages. The details of our parallelization schemes are elaborated in the next section.

### Multi-GPU-based parallelization designs

In this section we elaborate on the design of our novel parallelization model and PtyGer tool. We start with the introduction of a general parallelization design and discuss its limitations for the CG solver. We then present our hybrid parallelization model that can overcome the two challenges of CG solver parallelization.

#### General parallelization design

A general multi-GPU parallelization design consists of ***workload distribution*** and ***inter-GPU communication***, which has been employed by some existing works, for example, PtychoLib^40^. The objectives of these two components are performing diffraction pattern distribution and subimage border exchanges between GPUs, respectively.

**Figure 8:**
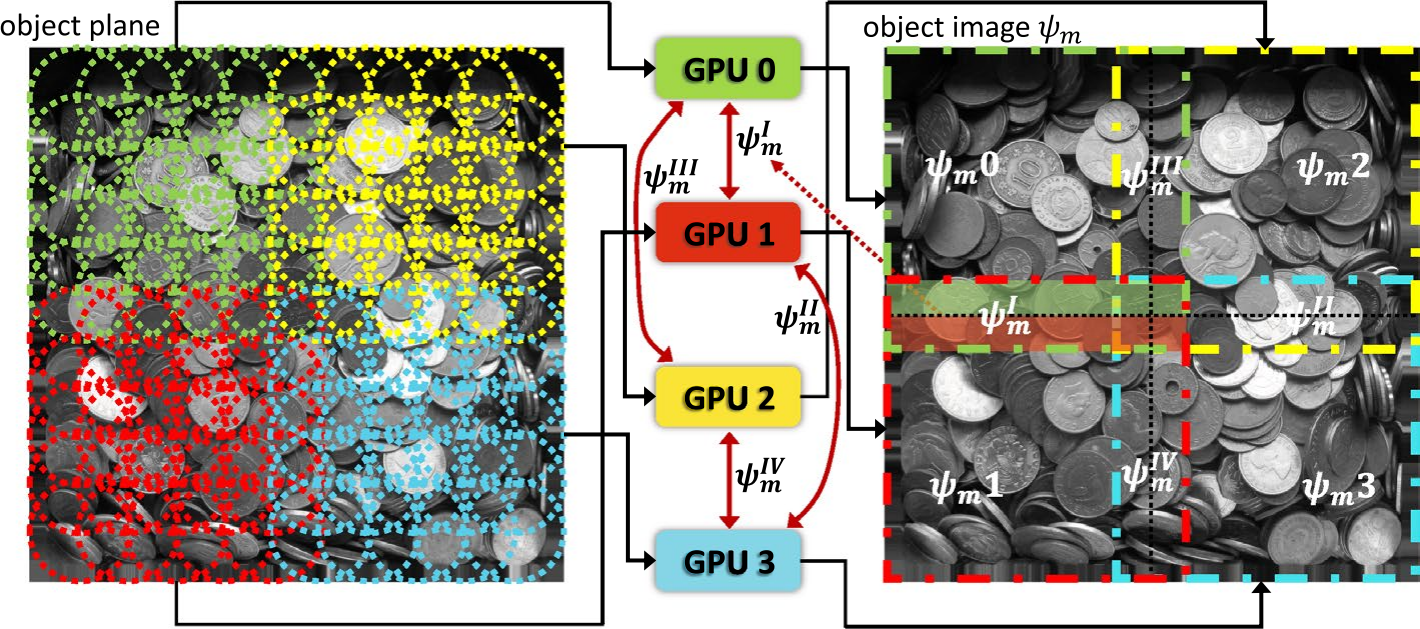
Basic parallelization design for a 4-GPU platform. The left-hand side of the GPUs illustrates the workload distribution while the right-hand side demonstrates the inter-GPU subimage border exchange. Each GPU stores a portion of the diffraction patterns and locally reconstructs the corresponding subimage. Neighbor GPUs exchange the subimage borders after each iteration.

##### Workload distribution

In ptychography, the size of the diffraction patterns *d* defines the workload size. Suppose that the detector is $512 \times 512$ and the number of scan positions is 16K. Then *d* requires $512^2\cdot 16K\cdot 4=16$ GB of memory space. This memory requirement, together with other data structures needed for reconstruction, can easily exceed the available memory in commodity GPUs. Therefore, we partition the diffraction patterns *d* and distribute them to many GPUs. The left-hand side of Fig. 8 schematically demonstrates the workload distribution scheme. On the left object plane, each circle represents a scan position and has a corresponding diffraction pattern (omitted in the figure for clarity). The diffraction patterns are divided and distributed to the GPUs, then each GPU locally reconstructs the corresponding subimage as shown on the right object image. For example, the diffraction patterns collected at green scan positions are stored in GPU 0; hence GPU 0 can locally reconstruct subimage $\psi _m0$. Notice that to keep the subimage borders accurate, in each GPU we store additional diffraction patterns at the halo of the subimage^73^, and we exchange the subimage borders with neighbor GPUs after each iteration.

##### Inter-GPU communication

To handle the border blurring, each GPU reconstructs also the halo of the corresponding subimage; hence, it has overlaps with its neighbor GPUs. The right-hand side of Fig. 8 shows how the subimages intersect with their neighbors. For each subimage, local reconstructions guarantee only that the central area (the area within the black dotted lines) is accurate. Its subimage halos are blurred because of the missed diffraction patterns stored in the neighbor GPUs. Therefore, the border areas of the sub-mages should be exchanged between GPUs after each object-update iteration to compensate for the halo blurring. For instance, GPU 0 and GPU 1 reconstruct two subareas enclosed by the green and red dashed squares, respectively. After each object update, the reconstructed subimages $\psi _m0$ and $\psi _m1$ are accurate compared with the whole image update. However, the overlapped area $\psi _m1$ is inaccurate in both GPUs. Specifically, the green area is part of $\psi _m1$’s halo and is blurred in GPU 1 while the red area is blurred in GPU 0. To make the halos accurate, GPU 0 then transfers the accurate green area to GPU 1 and receives the accurate red area transferred from GPU 1.

The general parallelization of ptychography can be summarized as follows. It partitions the diffraction patterns and distributes them to different GPUs in accordance with the GPU capacity. At each iteration, GPUs locally reconstruct the corresponding subimages with the halos, then synchronize and exchange the borders with their neighbor GPUs. After a certain number of iterations or reaching a stop criterion, we align the locally reconstructed subimages to obtain the entire object image.

#### Challenges of CG solver parallelization

Although the general parallelization is fully compatible with some reconstruction algorithms, for example, ePIE and the GD solver (Eq. (4) is directly applicable to partial reconstructions), it fails to preserve the CG solver’s algorithmic equivalence because of two main challenges.

##### Challenge 1

In each iteration, the CG solver computes the search direction $\eta _{m}$ using Eq. (6). Since $\eta$ and $\nabla _\psi F(\psi )$ are matrices that have the same shape as the object image, the partial processing is compatible with the first term of Eq. (6). However, the partial processing is not directly applicable to the second term’s coefficient computation.

We denote the coefficient as

8 $$\begin{aligned} \begin{aligned} \alpha _{m}=\frac{\left\Vert \nabla _\psi F(\psi _{m})\right\Vert _2^2}{ \langle \eta _{m-1},{\nabla _\psi F(\psi _{m})}-{\nabla _\psi F(\psi _{m-1})\rangle }}. \end{aligned} \end{aligned}$$

It is a scalar generated from the matrices $\nabla _\psi F(\psi _{m})$, $\nabla _\psi F(\psi _{m-1})$, and $\eta _{m-1}$ through norm $\left\Vert *\right\Vert$ and elementwise summation. Such matrices-to-scalar conversion implies that Eq. (8) demands the complete data of $\nabla _\psi F(\psi )$ and $\eta$, and therefore it is incompatible with the local partial reconstruction in the basic parallelization design. Specifically, in the running example, the local reconstructions of the four GPUs generate four distinct scalars $\alpha _m$0 to $\alpha _m$3 based on the partial $\nabla _\psi F(\psi _{m})$0 to $\nabla _\psi F(\psi _{m})$3 and $\eta _{m-1}$0 to $\eta _{m-1}$3, respectively. Neither of the four scalars can guarantee the convergence of the whole problem.

##### Challenge 2

Line search stage in the CG solver is a *while loop* that dynamical determines step length. It repeatedly computes the minimization function Eq. (2) with shrunken $\gamma _{m}$ until the condition Eq. (7) is fulfilled. In Eq. (2), *d* and ${{\,\mathrm{\mathscr {G}}\,}}\psi$ at the right-hand side are 3D arrays, while $F(\psi )$ at the left-hand side is a scalar. The array-to-scalar conversion is performed through the elementwise summation. Such dimensional reduction implies that computing $F(\psi _m+\gamma _m\eta _m)$ demands the complete data of *d* and ${{\,\mathrm{\mathscr {G}}\,}}\psi$. Therefore, similar to Eq. (8), the line search using Eq. (7) and Eq. (2) is incompatible with the local partial reconstruction in the basic parallelization design. With the basic design, different GPUs will generate different scalar $\gamma _{m}$, and none of them can guarantee the convergence of the whole problem.

Addressing this challenge requires a fine-grained parallelization with additional synchronization between GPUs for the CG solver. The advanced solution needs to be compatible with both Eqs. (2) and (6).

#### Hybrid model design

In our design, we propose a *hybrid parallelization model* for the CG solver aiming to fully maintain its algorithmic equivalence. As shown in Fig. 7, an iteration of the CG solver can be categorized in four stages. In the *GRAD stage*, the solver computes the gradient $\nabla _\psi F(\psi _{m})$ via Eq. (3). It then computes the search direction $\eta _{m}$ using Eq. (6) in the *DIR stage* and the step length $\gamma _{m}$ using line search in the *LS stage*. It updates the estimated object image $\psi _{m}$ with Eq. (5) in the *update stage*. The general parallelization design is applicable to the GRAD and update stages. To address the two challenges described in the preceding subsection, we use the ***gather-scatter*** communication pattern for the DIR stage and the ***all-reduce*** pattern for the LS stage. We couple the two patterns with the basic parallelization design to construct the hybrid model.

**Figure 9:**
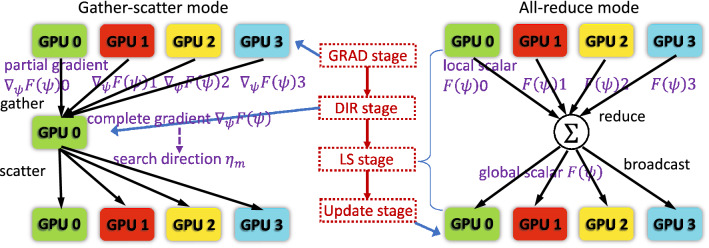
Schematic diagram of our hybrid parallelization model. The left drawing illustrates the workflow of gather-scatter mode while the right drawing demonstrates the workflow of all-reduce mode. The gather-scatter mode is dedicated to the DIR stage. The all-reduce mode is used to repeatedly compute the minimization function for the LS stage.

Figure 9 is a schematic depicting the hybrid parallelization model. The left part of the figure demonstrates the structure and workflow of the ***gather-scatter mode***, while the right part demonstrates the ***all-reduce mode***’s.

***Gather-scatter mode*** This mode is dedicated for the DIR stage. In this mode, one GPU is selected as the *master GPU*. Without loss of generality, in the running example, GPU 0 is used as the *master GPU*. In the GRAD stage, the perspective partial gradients $\nabla _\psi F(\psi _{m})$0 to $\nabla _\psi F(\psi _{m})$3 are computed and locally stored in the corresponding GPUs. Then in the DIR stage, all local partial gradients are gathered by the master GPU 0 to form the complete $\nabla _\psi F(\psi _{m})$. The complete gradient $\nabla _\psi F(\psi _{m})$ is a 2D matrix having the same shape of the object image $\psi _{m}$. Its data volume (usually smaller than $2048^2$) is far less than the 3D array diffraction pattern *d* that consumes most of the memory. Therefore, the gathering overhead is lightweight. Moreover, since $\nabla _\psi F(\psi _{m-1})$, $\eta _{m-1}$, and $\nabla _\psi F(\psi _{m})$ are all 2D matrices that can fit into a single GPU (GPU 0) and Eq. (6) does not rely upon 3D data *d* or ${{\,\mathrm{\mathscr {G}}\,}}\psi$, the complete search direction $\eta _{m}$ can be computed entirely in the master GPU 0. Then GPU 0 splits $\eta _{m}$ as $\eta _{m}$0 to $\eta _{m}$3 and scatters them to the corresponding GPUs for the next stage.

***All-reduce mode*** This mode is designed for the LS stage to overcome Challenge 2. As described in the preceding subsection, Eq. (2) requires the complete 3D arrays *d* and ${{\,\mathrm{\mathscr {G}}\,}}\psi$. In contrast to the 2D array $\nabla _\psi F(\psi _{m})$, however, gathering the partial *d* and ${{\,\mathrm{\mathscr {G}}\,}}\psi$ into a master GPU is infeasible because of their large data volumes. On the other hand, we observe that the outmost elementwise summation in Eq. (2) converts the 3D arrays to a scalar and is the only obstacle to the local partial reconstruction. Therefore, according to addition associativity, we can overcome Challenge 2 by summing up the partial results to get the global scalar $F(\psi _m+\gamma _m\eta _m)$. For instance, in the running example, the four GPUs compute four local scalars $F(\psi _m+\gamma _m\eta _m)$0 to $F(\psi _m+\gamma _m\eta _m)$3 via Eq. (2) using their perspective partial data *d*0 to *d*3, ${{\,\mathrm{\mathscr {G}}\,}}\psi _m$0 to ${{\,\mathrm{\mathscr {G}}\,}}\psi _m$3, $\psi _m$0 to $\psi _m$3, and $\eta _m$0 to $\eta _m$3. The global scalar $F(\psi _m+\gamma _m\eta _m)$ then can be calculated by $\sum _{g=0}^{3}F(\psi _m+\gamma _m\eta _m)g$ and broadcast to all GPUs. Here $\sum _{g=0}^{3}$ serves as the generalized reduction operation.

In summary, our proposed hybrid parallelization model utilizes the gather-scatter mode to overcome Challenge 1 by avoiding the computing with partial data. It gathers the partial data into a master GPU and performs the entire DIR stage in this GPU. The computed complete search direction is then divided and scattered to the corresponding GPUs. Our hybrid model employs the all-reduce mode to overcome Challenge 2 by reducing the partial results via a summation. Summing up each GPU’s partial result preserves the algorithmic equivalence to Eq. (2). The summed scalar $F(\psi _m+\gamma _m\eta _m)$ is then broadcast to all GPUs. The LS stage keeps iterating by shrinking the step length $\gamma _m$ and computing $F(\psi _m+\gamma _m\eta _m)$ in all-reduce mode until Eq. (7) is fulfilled. The hybrid model then switches back to the basic parallelization design and updates the object image $\psi _m$ accordingly.

### PtyGer implementation

We implement the proposed hybrid parallelization model in PtyGer, which is a high-performance multi-GPU based tool for ptychographic reconstruction. To conform to the computational imaging domain’s preference, PtyGer is written in Python using the CuPy (https://cupy.chainer.org/) library. Algorithm 1 demonstrates the complete workflow of PtyGer. After partitioning and copying the inputs from the host to the GPU devices (Line 4), PtyGer iteratively updates the object image until the number of iteration reaches the limit (Line 6–26). In each iteration, PtyGer first locally computes the partial gradients in the corresponding GPUs during the GRAD stage (Line 8–9), then switches to the gather-scatter mode to calculate the search-direction in the DIR stage (Line 10–14). It subsequently switches to the all-reduce mode and determines the step length via a line search (Line 15–23). During the line search, it repeatedly executes the minimization function (Line 19–22) until the stop condition is satisfied (Line 23). PtyGer then updates the subimages in every GPUs (Line 25) and exchanges their borders (Line 26). At the end of the iterative updates, all subimages are transferred from GPUs to CPU and aligned to form the complete object image (Line 27). PtyGer is open source and available at GitHub (https://github.com/xiaodong-yu/PtyGer).


